# Wirelessly Interrogated, Implantable Capacitive MEMS Sensors for Continuous Intraocular Pressure Monitoring

**DOI:** 10.3390/s26092806

**Published:** 2026-04-30

**Authors:** Liguan Li, Adnan Zaman, Ramesh Ayyala, Jing Wang

**Affiliations:** 1 Department of Electrical Engineering, University of South Florida, 4202 E. Fowler Avenue, Tampa, FL 33620, USA; 2 Microelectronics and Semiconductor Institute, King Abdulaziz City for Science and Technology (KACST), Riyadh 11442, Saudi Arabia; azaman@kacst.gov.sa; 3 College of Medicine Ophthalmology, University of South Florida, 13330 USF Laurel Dr 4th Floor, Tampa, FL 33612, USA; rayyala@usf.edu

**Keywords:** glaucoma, intraocular pressure sensor, LC tank circuit, mutual coupling, on-chip spiral inductor, variable capacitor, external readout circuit, telemetry

## Abstract

This work presents wirelessly interrogated microelectromechanical system (MEMS) capacitive sensors for continuous intraocular pressure (IOP) monitoring. The sensor uses a passive inductor–capacitor (LC) tank circuit comprising a fixed, on-chip spiral inductor and a pressure-sensitive, variable-gap capacitor with parallel-plate membrane electrodes and side anchors. The membrane is designed with dimensions of 500 µm × 500 µm × 2 µm and a capacitive transducer gap of 2.5 µm. Applied pressure deflects the top membrane, producing a corresponding capacitance variation that changes the frequency and phase response of the LC tank circuit, enabling real-time and continuous IOP monitoring over a target detection range of 0–50 mmHg and beyond. Mutual inductive coupling between the sensor and the external readout coil is investigated as a reliable readout mechanism.

## 1. Introduction

Intraocular pressure (IOP) is the fluid pressure within the eye, primarily determined by the balance between aqueous humor production and outflow in the anterior and posterior chambers. Maintaining normal IOP is essential for ocular structure and visual function. Abnormal IOP, particularly elevated IOP, is a major risk factor for glaucoma, a leading cause of blindness worldwide [1,2,3,4]. Glaucoma is often called the “silent thief of sight” because it usually progresses without symptoms until substantial, irreversible vision loss. It is characterized by progressive optic nerve damage and is usually associated with elevated IOP. As the second leading cause of visual impairment and blindness in the world, glaucoma affects approximately 1 in 40 adults over 40 years of age [5]. Current treatments aim to lower IOP through medications, laser therapy, or surgery. Among surgical options, trabeculectomy remains a standard procedure, particularly for moderate-to-severe glaucoma, by lowering IOP through reduced aqueous humor inflow or enhanced outflow.

Over the past decade, minimally invasive glaucoma surgery (MIGS) devices have emerged as a new class of Food and Drug Administration (FDA)-approved glaucoma treatments. Intended mainly for mild-to-moderate glaucoma, MIGS devices enhance physiologic aqueous humor outflow while minimizing tissue disruption. Building on the success of drainage implants in refractory glaucoma, clinicians have increasingly explored their use for earlier-stage surgical management. Randomized clinical trials suggest glaucoma drainage implants may offer advantages over trabeculectomy in selected cases and are gaining acceptance as the primary surgical option [6]. Their adoption has been supported by growing clinical experience and improved surgical techniques. In the United States, the annual number of MIGS-related procedures has remained relatively stable at 55,000 to 61,000 since 2003 [7]. From 1994 to 2012, trabeculectomy use declined by 77%, whereas glaucoma drainage implants increased by 410%, changing the trabeculectomy-to-drainage implant ratio from 27:1 in 1994 to 3:2 in 2012 [7]. According to the National Institutes of Health (NIH), about 120,000 Americans experience glaucoma-related vision loss, representing 9–12% of blindness in the United States. The global glaucoma population grew from 60.5 million in 2010 [8] to 76 million in 2020 and is projected to exceed 110 million by 2040 [9,10]. Despite the benefits of surgical intervention, long-term outcomes may be limited by postoperative fibrosis and scar formation that can lead to renewed IOP elevation. Continuous IOP monitoring is therefore essential for reducing the risk of progressive optic nerve damage associated with sustained IOP elevation.

Traditional IOP assessment methods, such as the Goldmann applanation tonometry (GAT) [11,12,13], noncontact tonometry (NCT) [13], and dynamic contour tonometry (DCT) [14], are widely used in ophthalmology. However, these techniques share a fundamental limitation: they provide only single-point IOP measurements, typically during daytime clinical visits. As a result, they fail to capture clinically relevant IOP fluctuations occurring throughout the day and night [15,16,17,18]. Increasing evidence indicates that IOP is dynamic rather than static, with circadian variations and significant short-term fluctuations. Peak values often occur outside regular clinic hours [16]. These unmonitored pressure spikes have been strongly linked to accelerated glaucomatous progression, even in patients whose daytime IOP remains within the target normal range [18,19,20].

Recent advances in sensing and telemetry have made continuous, non-invasive, and real-time IOP monitoring increasingly feasible, enabling characterization of IOP fluctuations with much higher fidelity than conventional episodic measurements. This progress represents a shift from isolated clinical “snapshots” toward a more comprehensive and dynamic understanding of ocular physiology. Emerging IOP monitoring platforms include wearable devices [21,22,23,24] and implantable telemetric sensors [25,26,27,28,29], each with distinct technical and clinical advantages. Continuous IOP monitoring could transform glaucoma management by enabling earlier detection of disease progression, more-accurate evaluation of treatment efficacy, and more-personalized care. At-home or real-time monitoring may also improve patient engagement and support more-proactive health decisions. In addition, remote monitoring can help clinicians intervene earlier, thereby reducing the risk of irreversible optic nerve damage.

In this work, a wirelessly interrogated MEMS-based capacitive pressure sensor is designed and implemented for continuous IOP monitoring. The sensor integrates a variable-gap capacitor with a fixed spiral inductor to form a passive LC resonant circuit. Pressure-induced capacitance changes shift the resonant frequency, which is detected through mutual inductive coupling with an external readout unit. The sensor is designed for a pressure range of 0–50 mmHg, covering both the normal IOP range of approximately 10–21 mmHg [30] and the broader abnormal range relevant to glaucoma monitoring. The sensor structure is modeled in CoventorWare 10.2 using finite element analysis, with membrane geometry and material properties optimized for sensitivity and mechanical robustness. Based on the design results, electroplated nickel is selected as the structural material for the suspended membrane. An equivalent circuit model of a co-fabricated, on-chip spiral inductor is incorporated to realize a battery-less LC tank circuit coupled inductively to an external readout coil. This entire implantable sensor and readout system is further analyzed in Keysight Advanced Design System (ADS) 2023. A benchtop prototype based on the same LC resonant coupling principle is also implemented to evaluate amplitude- and phase-based readout schemes at a target interrogation distance of approximately 4 cm, thus supporting practical point-of-care use by end users for glaucoma monitoring.

## 2. Materials and Methods

The intraocular pressure (IOP) sensor consists of a fixed spiral inductor and a variable capacitor that together form a passive LC tank circuit. The capacitor has two parallel plates: a fixed bottom electrode and a movable, pressure-sensitive membrane acting as the top electrode. When subjected to intraocular pressure, the membrane deflects, altering the air gap between the electrodes and thus changing the capacitance. A smaller initial air gap is expected to produce a higher baseline capacitance and a larger capacitance variation across the target pressure range of 0–50 mmHg. These pressure-induced changes in the LC tank circuit appear as shifts in resonant frequency and phase response that can be measured wirelessly by an external readout circuit and a vector network analyzer (VNA).

**Figure 1:**
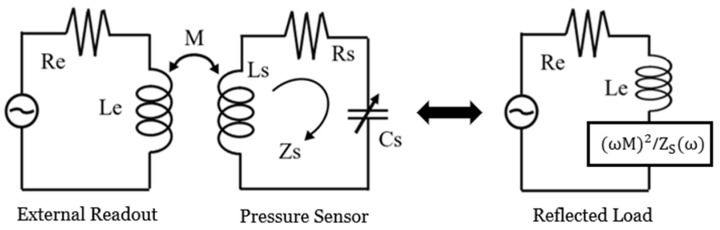
Equivalent circuit of an intraocular pressure sensor with an inductively coupled readout circuit, which converts the sensor’s response into an additional reflected load impedance at the external readout coil and is detected as a complex impedance near the sensor’s resonant frequency.

Figure 1 presents the equivalent circuit model of the intraocular pressure sensor with an inductive coupling link for telemetric readout. The sensor’s LC tank is modeled using a fixed inductor (*L*s), a series resistor (*R*s), and a variable capacitor (*C*s). The resonant frequency (*f*_0_) of this sensor is given by [31]

(1) $$f_{0}=\frac{1}{2\pi \sqrt{LsCs}}$$

As the applied pressure changes, the capacitance *C*s varies accordingly, resulting in a shift in the resonant frequency. This frequency shift enables wireless pressure sensing through inductive telemetry with an external readout coil. Through magnetic coupling, the external coil excites the LC resonator of the implantable IOP sensor and supports wireless readout. The mutual inductance between the sensor and readout coils is given by

(2) $$M=k\sqrt{LeLs}$$

(3) $$k=\frac{1}{{\left[1+2^{\frac{2}{3}}{\left(\frac{d}{\sqrt{r_{1}r_{2}}}\right)}^{2}\right]}^{\frac{3}{2}}}$$

where the mutual inductance *M* is defined as the proportionality between the electromotive force (EMF) generated in coil 2 (readout coil) to the time-varying current in coil 1 (sensor coil). Here, *k* is the coupling coefficient; *L*e is the external readout coil inductance; and *r*_1_ and *r*_2_ are the radii of the external readout coil and the on-chip spiral inductor integrated with the sensor, respectively [32].

The sensor’s resonant frequency is detected by monitoring the change in the total impedance of the external coil caused by the reflected load of the sensor. The peak magnitude of the corresponding impedance phase dip can be expressed as follows [31]:

(4) $$\Delta \varphi_{DIP}≅{tan}^{-1}(\frac{\omega_{0}M^{2}}{LeRs})$$

where *ω*_0_ is the angular resonant frequency; *R*s is a series resistance of the sensor; *M* is the mutual inductance; and *L*e is the inductance of the external readout coil.

To maximize the phase dip magnitude, the series resistance (*R*s) of the electroplated inductor should be minimized while the inductance (*L*s) should be increased, with a sufficiently strong mutual inductance (*M*). This involves a key trade-off. Increasing *L*s by adding more coil turns also increases parasitic capacitance, which lowers the self-resonant frequency of the planar inductor. Thus, proper device operation requires the inductor self-resonant frequency (*f*_0_) to remain well above the operating frequency (*f*_op_). Minimizing *Rs* is critical for improving resonant frequency detection resolution. In practice, low-resistance, high-*Q* inductors are typically realized using thick, low-resistivity metal layers.

### 2.1. MEMS Membrane Capacitor Design

The MEMS membrane capacitors were designed and analyzed using CoventorWare. This simulation study first evaluated candidate structural materials and membrane geometries. Nickel, copper and silicon were considered in the finite element method (FEM) simulations. Nickel was ultimately chosen as the structural material for the MEMS capacitive membrane due to its biocompatibility and nonmagnetic behavior, which helps avoid interference with nearby sensitive electronics. Copper was selected for the bottom electrode because of its high electrical conductivity. During the MEMS capacitor design, a broad range of membrane dimensions, thicknesses, and air-gap spacings was parametrically investigated. In parallel with the CoventorWare FEM analysis, closed-form analytical calculations were performed. The expressions for the maximum displacement and capacitance are given by the following [33]:

(5) $$W_{max}=0.00133\frac{pl^{4}}{D}$$

where $D=Et^{3}/(12\left(1-v^{2}\right))$ is the flexural rigidity; *p* is the applied pressure; *E* is the Young’s modulus; *ν* is the Poisson’s ratio; *t* is the thickness of the top membrane; and *l* is the top-membrane length.

(6) $$C=C_{0}(1+\frac{12.5pl^{4}}{2025Dt})$$

where *C*_0_ = *εA/d*, and *d* is the gap spacing between two capacitor electrodes.

When external pressure is applied to the top membrane, the membrane deflects and changes the capacitance. This pressure-induced capacitance variation shifts the resonant frequency, as described in Equation (1). Designs in which the calculated maximum membrane displacement exceeded the air-gap spacing were excluded because they could cause electrode stiction and short circuiting. The remaining designs, featuring different membrane geometries but identical membrane thickness and air-gap spacing, were then compared systematically.

Comparative results for three membrane sizes, each with a membrane thickness of 2 µm and an air gap of 2 µm, are presented below. As expected, the analytical and simulated results diverge more noticeably at higher IOP levels. This discrepancy occurs because the FEM model captures nonlinear deformation and other physical effects, including Poisson’s ratio effects, more accurately than the simplified analytical formulation.

**Figure 2:**
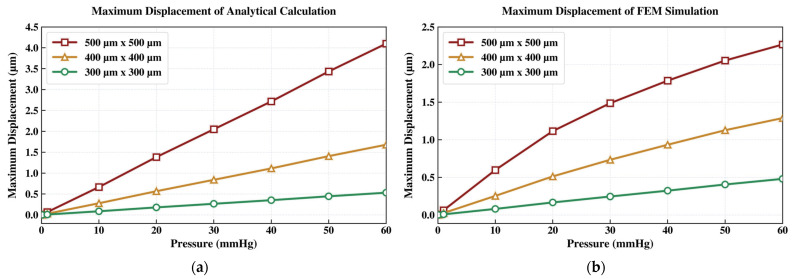
Comparative study of the maximum membrane displacement vs. pressure, determined by: (**a**) analytical calculations and (**b**) FEM simulation.

As shown in Figure 2, the maximum membrane displacement increases with the applied pressure for all designs when the membrane thickness and air gap are fixed at 2 µm. The 300 µm × 300 µm membrane (green curve) shows the smallest displacement because of its relatively high structural stiffness. Both the analytical model and FEM simulation indicate that the peak displacement remains limited to approximately 0.5 µm at 60 mmHg, well below the 2 µm air gap. Although the analytically calculated capacitance (0.4292 pF) and FEM-simulated value (0.4462 pF) at 60 mmHg are reasonable consistent, the total capacitance variation over the target IOP range is only about 0.03 pF. This small variation may be challenging for robust electronic detection.

**Figure 3:**
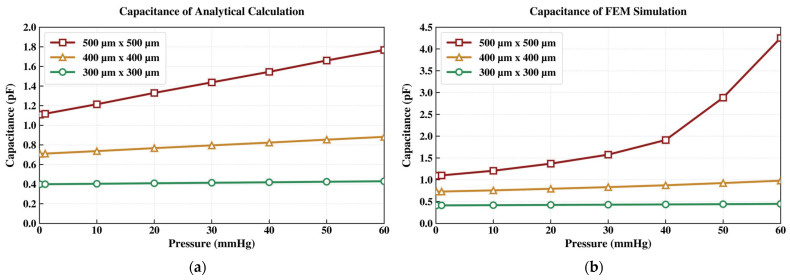
The capacitance vs. applied pressure response derived from: (**a**) analytical modeling and (**b**) finite element method (FEM) simulation.

The 400 µm × 400 µm membrane design (orange curve) provides greater compliance and therefore larger pressure-induced deflection. As shown in Figure 2b, the FEM simulation predicts a maximum displacement of 1.2876 µm at 60 mmHg, significantly greater than that of the 300 µm × 300 µm design. Correspondingly, the capacitance increases from 0.7274 pF to 0.9789 pF, as shown in Figure 3b. Although this design yields a larger capacitance change, the resulting signal magnitude may still be challenging for reliable readout.

Increasing the membrane size to 500 µm × 500 µm (red curve) further improves sensitivity but violates the air-gap constraint. As shown in Figure 2b, the FEM simulation predicts a peak membrane displacement of 2.2661 µm at 60 mmHg, exceeding the initial 2 µm air gap and indicating likely contact between the capacitor plates. This interpretation is also supported by the abrupt change in the FEM-simulated capacitance curve above 50 mmHg in Figure 3b, which is consistent with electrode collapse under high pressure.

To prevent contact between the capacitive membrane and the bottom electrode, two design strategies were evaluated. The first strategy was to increase the membrane thickness while keeping the air gap fixed. The second was to increase the air-gap spacing while retaining a membrane thickness of 2 µm. The results showed that increasing membrane thickness also increased structural stiffness, which reduced pressure sensitivity. Thus, final design optimization focused on adjusting the air gap to provide sufficient electrode clearance while avoiding mechanical collapse. A dielectric layer, such as HfO_2_, was introduced on the bottom electrode to protect against short circuiting.

The 500 µm × 500 µm membrane was selected because it yielded the largest membrane displacement and capacitance change. As seen previously in Figure 2 and Figure 3, the red curves correspond to this design with a 2 µm air gap. However, the parallel-plate electrodes were predicted to collapse before reaching the upper IOP range, suggesting the need for a modest increase in air-gap spacing.

**Figure 4:**
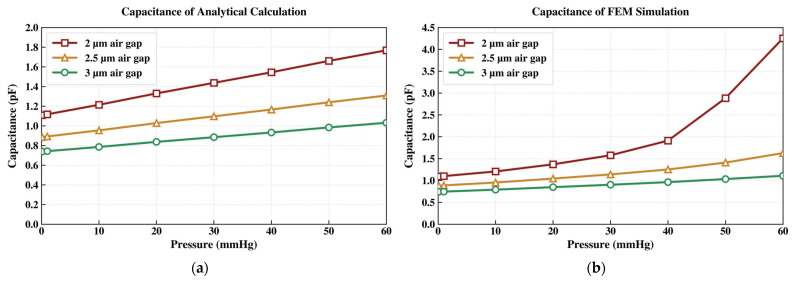
Capacitance vs. applied pressure characteristic derived from: (**a**) analytical modeling and (**b**) finite element method (FEM) simulation.

Increasing the air gap from 2 µm to 2.5 µm eliminates the electrode-collapse issue for the 500 µm × 500 µm design. Figure 4 compares this fixed membrane geometry, with a fixed membrane thickness of 2 µm, across different air-gap spacings.

### 2.2. Integrated Spiral Inductor Design

The integrated spiral inductor was designed and simulated using Momentum, a 2.5-dimensional (2.5-D) electromagnetic simulator in Keysight Advanced Design System (ADS). Nickel was selected as the inductor material because of its biocompatibility [34]. A square spiral geometry was adopted because it provides relatively high inductance while retaining compatible with standard IC layout constraints. Spiral inductors are widely used in RF integrated circuits (RFICs) because they can achieve relatively high inductance in a compact footprint. In general, increasing the number of turns increases the total conductor length and the effective inductance.

As discussed above, a longer spiral conductor increases the effective inductance. To maximize inductor length while efficiently using chip area, the spiral coil was arranged around the membrane capacitor. However, adding turns within a limited chip area also raises parasitic capacitance between adjacent turns, which can degrade inductor performance. The inductor design requires careful optimization of multiple geometric parameters, including the number of turns, line spacing, conductor width, and overall dimensions. The quality factor (*Q*) is especially important because it strongly affects the performance of the inductively coupled readout circuit. The inductance and quality factor are given by the following [35]:

(7) $$Ls=K_{1}\frac{\mu_{o}n^{2}D_{avg}}{1+K_{2}\rho }$$

(8) $$Q=\frac{2\pi f_{0}Ls}{Rs}$$

where $K_{1}$ and $K_{2}$ are layout-dependent coefficients. For a square-shaped spiral inductor, $K_{1}=2.34$ and $K_{2}=2.75$. *D*_avg_ is the average diameter, defined as *D*_avg_ = (*D*_out_ + *D*_in_)/2, and *ρ* is the fill ratio, defined as *ρ* = (*D*_out_ *− D*_in_)/(*D*_out_ + *D*_in_); *n* is number of turns; *f*_0_ is the operation frequency; and *μ*_o_ is the free-space magnetic permeability.

Nickel was selected to simplify the fabrication by allowing the inductor and other structural elements to be realized using a compatible material system. This choice is further supported by nickel’s established use in biomedical applications, its biocompatibility, and its nonmagnetic behavior, which avoids interference with nearby sensitive electronics. Although nickel has a higher resistivity than copper, simulation results confirmed that a sufficiently high *Q*-factor for inductive coupling can still be achieved through meticulous design. However, the *Q*-factor lowers gradually as the number of turns increases, mainly because the longer conductor length increases the series resistance in the electroplated nickel inductor.

As shown in Table 1, for a spiral inductor with a total length of 10,050 µm and a line width of 20 µm, the total number of squares used for sheet resistance calculation is 502.5, obtained by dividing the total conductor length by the line width. Given a tested inductor resistance of 41.55 Ω, the corresponding sheet resistance is 0.0827 Ω/□. The effective resistivity of the electroplated nickel is obtained by multiplying the sheet resistance by the nickel thickness, yielding 8.5 × 10^−8^ Ω·m. This value is slightly higher than the bulk nickel resistivity, 7.2 × 10^−8^ Ω·m, consistent with the anticipated properties of the electroplated nickel.

### 2.3. External Readout Coil Design

The readout coil inductor was fabricated by soldering a copper coil to a Subminiature A (SMA) connector, which interfaces with a vector network analyzer (VNA) to measure frequency-dependent phase and amplitude responses. The coil inductance is determined by geometric parameters such as total conductor length, number of turns, and wire diameter. Because adding turns increases the total conductor length, the inductance increases approximately in proportion to the turn count.

### 2.4. Fabrication Process of the IOP Sensor

**Figure 5:**
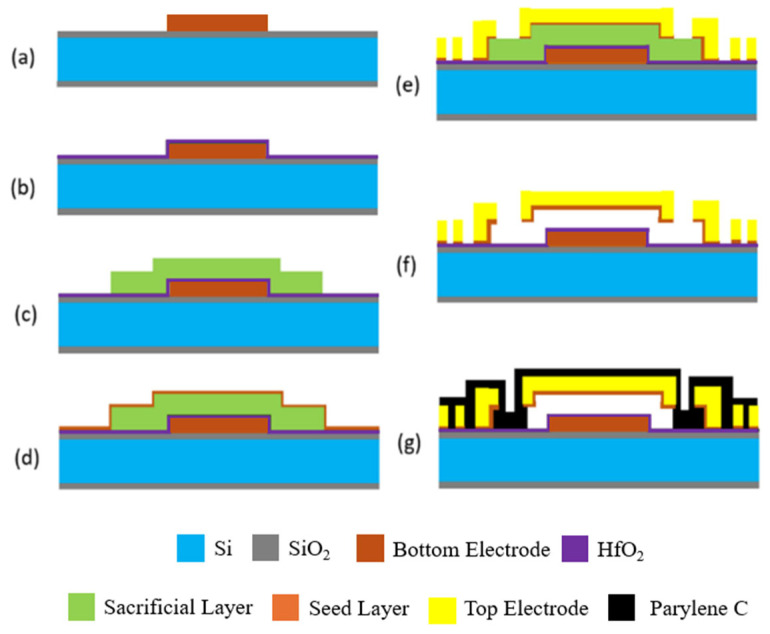
Step-by-step fabrication process of IOP sensor: (**a**) sensor bottom electrode formation via lift-off; (**b**) atomic layer deposition of an HfO_2_ insulating barrier to prevent short circuiting; (**c**) sacrificial layer formation; (**d**) seed layer sputtering for nickel electroplating; (**e**) nickel electroplating form the top electrode and spiral inductor; (**f**) device release by removing sacrificial layer; and (**g**) device hermetic sealing with Parylene C.

The complete fabrication process flow for the MEMS capacitive sensor is depicted in Figure 5. The process begins with thermal oxidation of a polished silicon substrate to form a 1.5 µm insulation film, which separates the surface micromachined structures from the underlying silicon. Next, a 200 nm Cr/Ru metal stack is defined by sputtering and lift-off to serve as the capacitor bottom electrode. A 120 nm HfO_2_ layer is then blanket-deposited over this bottom electrode as a dielectric safety shield to avoid short circuiting. A 2.5 µm thick photoresist layer is applied to act as the sacrificial layer. Nickel electrodeposition is then performed using a Ti/Cu seed layer to form the spiral inductor and the 2 µm thick capacitor top electrode simultaneously.

For nickel electroplating, a nickel sulfamate bath was selected to provide a deposited membrane with low residual strain and high ductility. Boric acid was added to maintain the pH between 3.5 and 4.5, suppressing pitting and morphological irregularities. Nickel chloride and sodium lauryl sulfate were added to bolster conductivity and improve brilliance, respectively. The wafer was connected to the cathode, while a nickel sheet acted as the anode. When a controlled current was applied to achieve desired current density and plating rate, Ni^2+^ cations were electrostatically drawn to and deposited on the cathode surface. After electroplating, the seed layer was selectively removed by wet etching.

For the final MEMS release process, the wafer is first diced. The diced chip is then immersed for two minutes in a 1:1 mixture of 30% H_2_O_2_ (Hydrogen Peroxide) and 29% NH_4_OH (Ammonium Hydroxide) to selectively strip the Ti/Cu seed layers from the field area. The sacrificial photoresist is then dissolved using AZ400T stripper or 1165 remover to release the capacitive membrane-based IOP sensor. Finally, a conformal Parylene C film is deposited over the completed structure to form a hermetic seal over the capacitive transducer.

**Figure 6:**
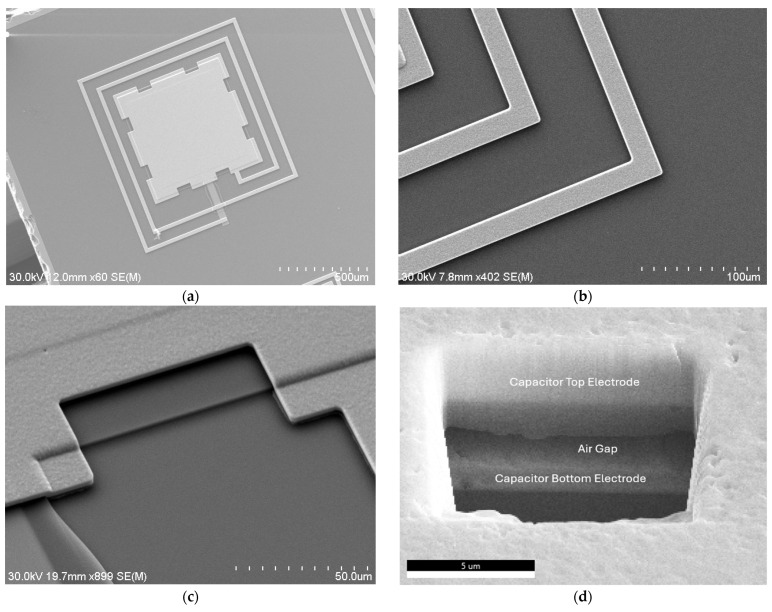
(**a**) SEM image of a microfabricated IOP sensor made of electroplated nickel; SEM photos of electrodeposited nickel layer, showing its (**b**) surface characteristics and (**c**) topographical uniformity; (**d**) FIB cross-section view of the air gap of a microfabricated and fully released IOP sensor.

Figure 6 shows the Hitachi SU-70 scanning electron microscopy (SEM) (Hitachi High Technologies America, Inc., Schaumburg, IL, USA) and focused ion beam (FIB) (Thermo Fisher Scientific Inc., Waltham, MA, USA) images of a fabricated IOP sensor. Several release openings are incorporated around the top-electrode periphery to facilitate device release. The HfO_2_ dielectric layer was deposited without needing a mask. Only four photomasks and lithography steps were needed to fabricate this IOP sensor, significantly reducing microfabrication complexity and processing time. Moreover, the achievable minimum alignment tolerance of up to 5 µm mitigates misalignment concerns. As shown in Figure 6d, the capacitor air gap is verified in a released device using FIB cross-sectioning. A cavity is milled at the center of the capacitive membrane to reveal the layer distribution, including the suspended air gap. The fully released IOP sensor is then hermetically sealed with Parylene C, which provides an excellent barrier with very low permeability to moisture and gases.

## 3. Results and Discussion

The wireless interrogated IOP sensor consists of a closed-loop spiral inductor connected to a micromachined capacitive membrane with an air gap. Deformation and possible stiction-induced collapse of the microfabricated air-gap capacitor are important because the sensor resonant frequency depends on the deformation of the pressure-sensitive top membrane. DC probing can be used to verify that the microfabricated capacitor remains open-circuited, indicating the absence of collapse or stiction. Also, one-port RF probing measurements are performed by a ground-signal-ground (GSG) probe to evaluate membrane deformation.

### 3.1. On-Chip Sensor Probing Measurement

**Figure 7:**
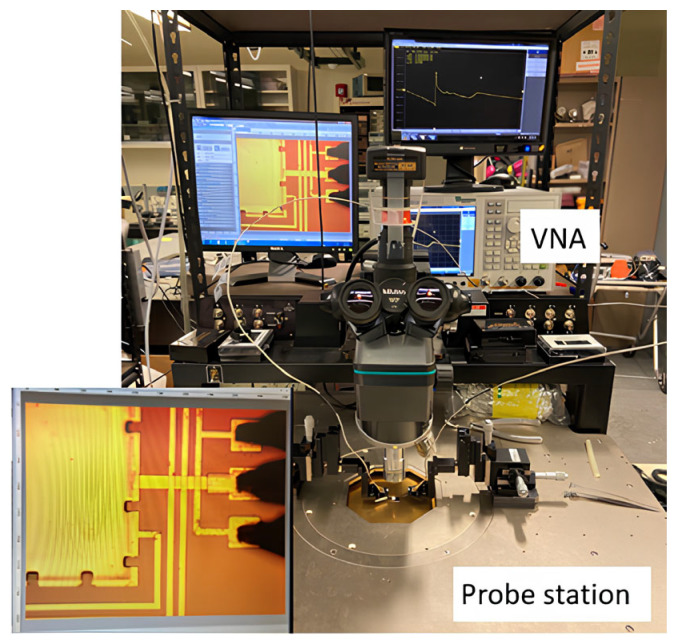
On-chip IOP sensor RF probing measurement setup with a VNA and a prober.

**Figure 8:**
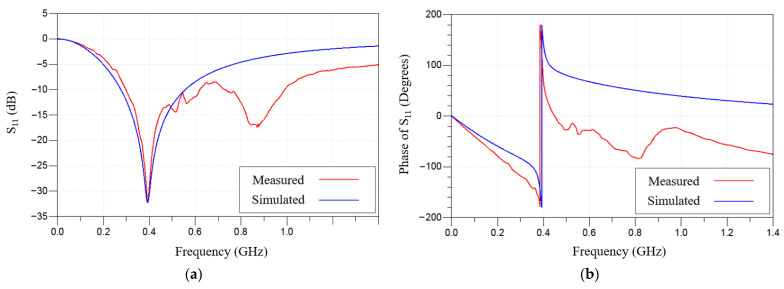
Measured (**a**) frequency and (**b**) phase responses of microfabricated on-chip IOP sensor.

The wirelessly interrogated IOP sensor consists of a closed-loop LC circuit formed by a series-connected inductor (L) and capacitor (C). On-chip RF probing measurement after the device microfabrication is shown in Figure 7. The microfabricated capacitor has a tendency to collapse during its releasing process (also known as a stiction issue), which is important because the resonant frequency depends on the deformation of the top pressure-sensitive membrane of the MEMS membrane-based capacitor. As shown, the series-connected inductor and MEMS capacitor in one-port configuration with a GSG probe is used for RF probing measurement. The amplitude and phase around the resonant frequency can be obtained using a Keysight E5071C vector network analyzer. As shown in Figure 8, the on-chip IOP sensor exhibits the resonant frequency values of 393.315 MHz and 394.159 MHz for simulation and measurement, respectively, accompanied by a reflection coefficient of −32 dB and an abrupt phase transition from −180 degrees to 180 degrees.

### 3.2. Wireless Pressure Sensor Measurement

**Figure 9:**
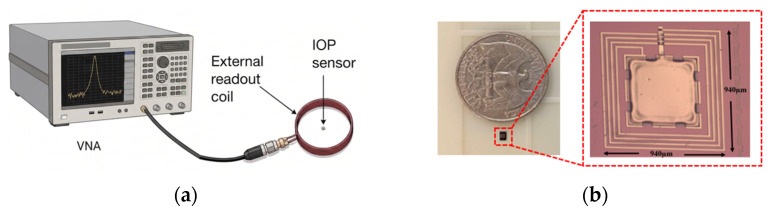
(**a**) Wireless pressure sensor measurement setup; (**b**) a fabricated IOP capacitive membrane sensor with an integrated spiral inductor, shown beside a U.S. quarter for size comparison.

**Figure 10:**
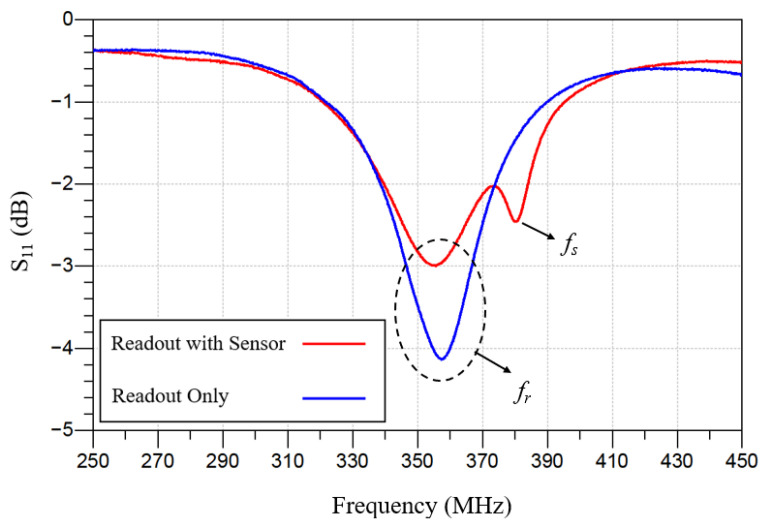
Frequency responses of the readout coil with and without the presence of the IOP sensor.

Figure 9a shows the wireless pressure sensor measurement setup used to characterize the sensor frequency response under zero applied pressure with an external interrogation coil. Through mutual inductive coupling between the external readout coil and the on-chip spiral inductor, the resonant response of the implantable MEMS IOP can be detected wirelessly. The readout coil is connected to a Keysight PXI M9372A vector network analyzer (VNA) to measure the RF response. Figure 9b shows a diced pressure sensor chip after device release and hermetic sealing with Parylene C. Individual IOP sensor devices obtained from wafer dicing were used for wireless telemetry characterization. Figure 10 shows the measured responses of the IOP sensor with an inductive readout. The blue curve shows the frequency response for the readout coil alone, while the red curve shows the measured frequency response in the presence of the IOP sensor.

**Figure 11:**
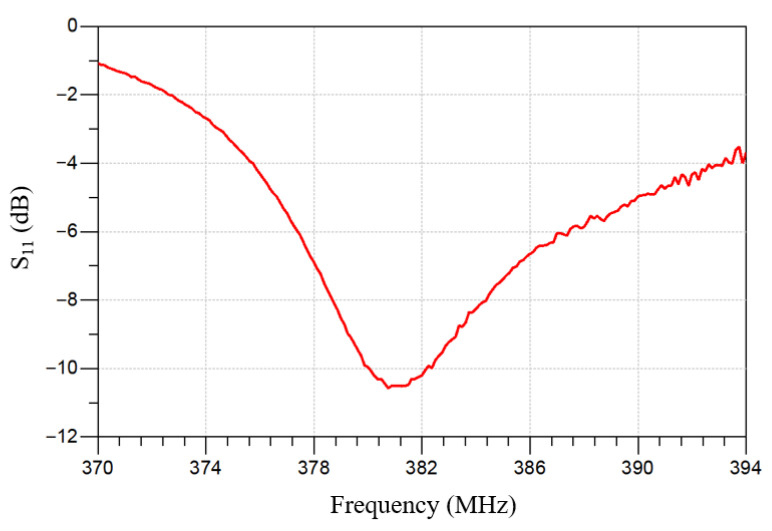
Measured frequency response of the IOP sensor using a vector network analyzer (VNA) after applying de-embedding technique between the measured frequency responses of the readout coil with and without inductive coupling to the sensor.

To extract the intrinsic response of the MEMS IOP sensor, a de-embedding procedure was used to remove measurement-setup effects. The measured frequency response agrees with the analytical model predicted using probe-measured inductance and capacitance values from identically sized, one-port inductor and capacitor test structures. As shown in Figure 11, the resonant frequency measured using a vector network analyzer (VNA) is 381.1 MHz, identical to the value predicted by Equation (1).

**Figure 12:**
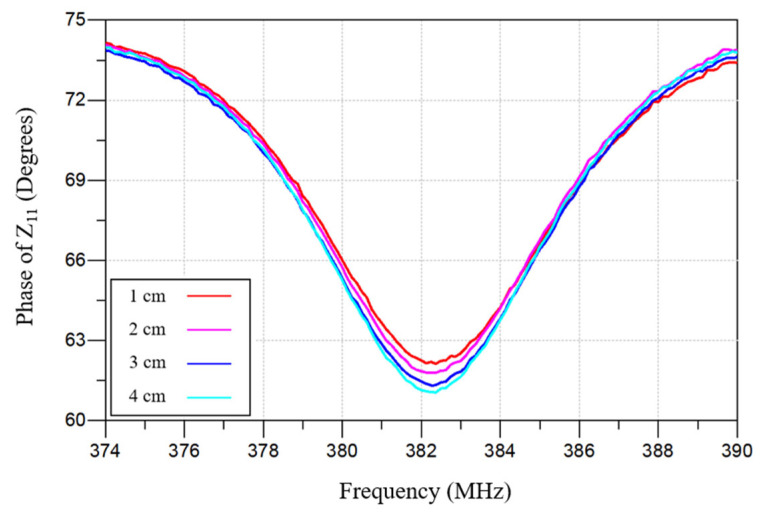
Measured phase dip vs. frequency responses of the IOP sensor using a vector network analyzer (VNA) while adjusting the IOP sensor-to-external readout coil distance from 1 cm to 4 cm.

A square spiral inductor was selected because it provides relatively high inductance. The key design parameters of the IOP sensor are listed in Table 2. To evaluate the effect of separation distance between the implantable IOP sensor and the external readout coil, wireless pressure sensor measurements were performed at distances from 1 cm to 4 cm as shown in Figure 12. Increasing the separation distance weakens the transmitted signal and impacts the phase dip, but it did not affect the resonant frequency.

### 3.3. Wireless Pressure Sensor Water Balloon Testing

**Figure 13:**
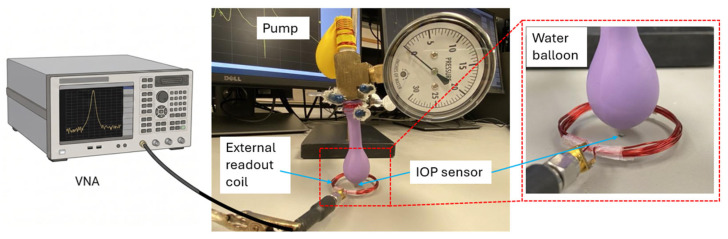
Schematic illustration of the IOP sensor water balloon testing setup, including a fluid pressure system (water balloon, pump, and reference sensor for pressure calibration) and the inductively coupled readout circuitry (a readout coil and a vector network analyzer).

An experimental pressure-loading setup, often referred to as the water balloon testing method, was used to characterize the microfabricated IOP sensor under controlled conditions. As depicted in Figure 13, water is pumped into a balloon to create a controlled pressure that simulates IOP, enabling pressure loading tests over the target IOP range of 0–50 mmHg. This pressure deforms the top membrane of the capacitive sensor, causing a capacitance change that shifts the resonant frequency of the LC sensor. A Keysight PXI M9372A vector network analyzer (VNA) performs frequency-sweep measurements and captures the corresponding magnitude and phase responses under prescribed hydrostatic liquid-pressure loads.

Figure 13 depicts the measurement setup that includes the water balloon and pump, a reference pressure sensor for calibration, an external readout coil, and the VNA. Through mutual inductive coupling between the external coil and the on-chip spiral inductor, the resonant response of the implantable IOP sensor can be detected wirelessly in a noncontact fashion. In this configuration, the external coil excites the implanted LC sensor, and the sensor reflects an impedance load back to the readout coil. By monitoring the resulting change in the external-coil impedance with the VNA, the resonant frequency shift and phase variation can be measured in real time without direct electrical contact. The sensor response is most clearly observed by tracking the phase of the external-coil impedance, which shows a distinct dip near resonance.

**Figure 14:**
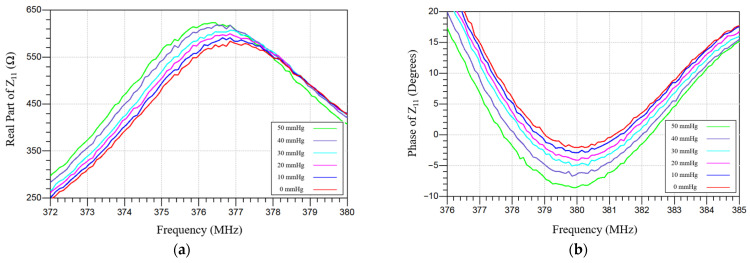
Measured (**a**) real part and (**b**) phase of impedance characteristics of the IOP sensor, acquired by using a VNA and an external readout coil. The induced frequency and phase shifts are directly proportional to the hydrostatic pressure load delivered by the water balloon setup.

**Figure 15:**
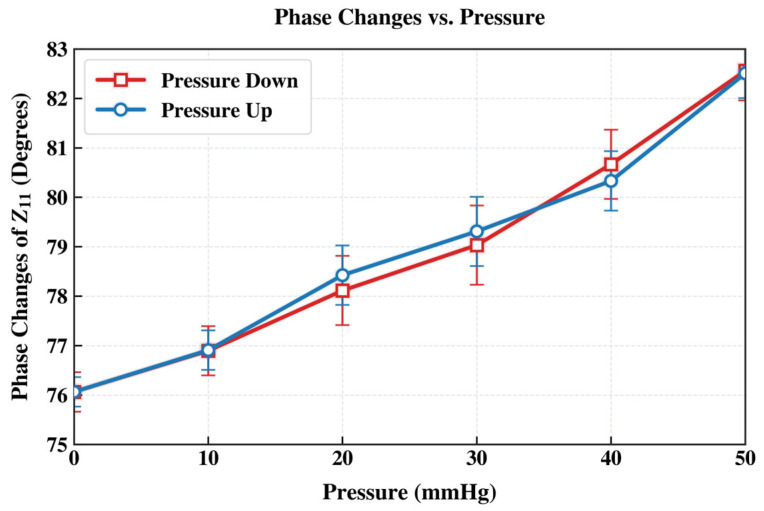
Extracted phase-dip changes across the hydrostatic liquid pressure load range of 0–50 mmHg. The observed pressure-induced decrease in peak phase dip magnitude is attributed to the increased membrane capacitance under the rising liquid pressure applied by the water balloon.

Figure 14 shows the measured real part of impedance and the phase dip-shift of the IOP sensor over a liquid pressure range of 0–50 mmHg. Figure 15 presents the measured peak magnitude of the phase dip over the same pressure region. The phase-dip magnitude is maximized when the series resistance (*R*s) of the integrated inductor is minimized. Although increasing the sensor’s inductance (*L*s) can enhance mutual coupling (*M*), augmenting *L*s by adding coil turns also increases parasitic capacitance. This lowers the self-resonant frequency and constrains the operational frequency range. In practice, the self-resonant frequency should remain at least three times higher than the intended operating frequency. Thus, minimizing the inductor series resistance (*R*s) is crucial for improving the detection limit (resolution) of the proposed IOP sensor. Low-*R*s, high-*Q* integrated spiral inductors can be achieved by using thicker, highly conductive metal layers formed by optimized, low-resistivity electroplating. The results indicate the performance could be improved through more-advanced wireless telemetry schemes, especially by improving resolution, repeatability, external-coil alignment tolerance, and calibration simplicity.

### 3.4. Wireless Pressure Sensor Underwater Testing

**Figure 16:**
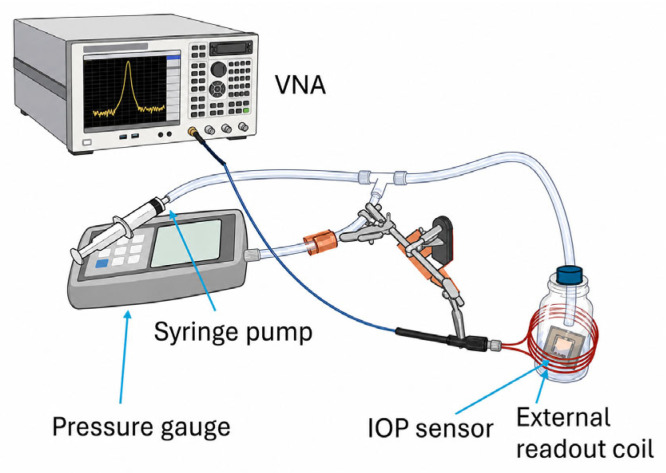
Schematic illustration of the custom-built IOP sensor underwater testing setup, featuring the fluid pressure system (a syringe pump and a pressure gauge), the IOP sensor submerged under the liquid environment, and the inductive readout system (a readout coil and a VNA).

In addition to the water balloon testing method, the microfabricated wireless capacitive IOP sensor was characterized by a custom-built underwater apparatus for controlling liquid pressure loading. As shown in Figure 16, this setup consists of a syringe pump, a pressure gauge, an IOP sensor, an external readout coil, and a Keysight PXI M9372A vector network analyzer (VNA). In the submerged configuration, the syringe pump applies controlled liquid pressure to simulate IOP while the sensor is submerged in the liquid. The VNA measures the corresponding sensor response in terms of resonant-frequency shift and phase variation.

**Figure 17:**
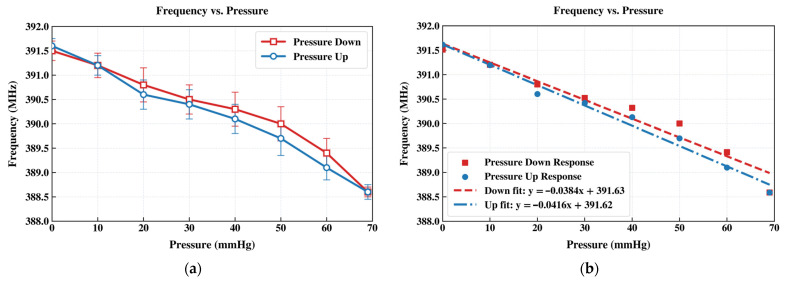
(**a**) Frequency-response curves of the IOP sensor obtained with an external interrogation coil and a VNA under hydrostatic pressure ranging from 0 to 70 mmHg. The decrease in resonant frequency with gradually increasing pressure confirms the increase in membrane capacitance. (**b**) Measured frequency responses under pressure-down and pressure-up procedures that are fitted against linearly extrapolated curves showing sensitivity values across the full range of 0–70 mmHg.

To mimic intraocular conditions, liquid was introduced into a glass vessel to create a controlled pressure environment, allowing characterization over a pressure range of 0–70 mmHg. The pressure in the custom-built apparatus was increased incrementally using a syringe pump, causing deflection of the sensor’s top membrane. This deformation alters the membrane capacitance and produces a corresponding resonant frequency shift. Due to the readout circuit design, both the magnitude and phase of the resonant response can be measured precisely using RF techniques. Swept-frequency measurements were therefore conducted at prescribed hydraulic pressure levels using a vector network analyzer (VNA). Figure 17a presents the measured frequency response of the IOP sensor, acquired through the external coil and a VNA, under applied liquid pressure from 0 to 70 mmHg. The results show a clear decrease in resonant frequency with increasing pressure, consistent with the expected increase in sensor capacitance induced by membrane deflection under applied pressure. Compared with the water balloon measurements conducted before Parylene C encapsulation, the parylene-coated IOP sensor intended for underwater operation exhibits a slightly higher resonant frequency, which can be largely ascribed to the additional parasitic loading introduced by the Parylene coating on the spiral inductor. Figure 17b depicts the linearly extrapolated IOP sensor pressure-induced frequency response curves showing sensitivity values across the full range of 0–70 mmHg.

### 3.5. Comparison with Other IOP Sensors

Table 3 summarizes previously reported implantable IOP sensors in terms of sensing mechanism, operating frequency, quality factor, device size, capacitance, inductance, and sensitivity extracted from underwater IOP sensor responses shown in Figure 17. The sensor developed in this work achieves the smallest device footprint among the compared designs. This compact size is needed to fit the sensor inside a cavity within an Ahmed glaucoma valve, an FDA-approved implant drainage device used in minimally invasive glaucoma surgery (MIGS). By targeting a particular Ahmed glaucoma valve (AGV) FDA-approved glaucoma drainage device or an intraocular lens (IOL), the sensor is diced to achieve a minimum size of 0.94 × 0.94 × 0.5 mm^3^ to adapt to either device for future in vivo study. Both approaches, when adopted in future in vivo study, are anticipated to offer minimal invasiveness, small size, biocompatibility, and long-term stability.

## 4. Conclusions

This work presented the design, modeling, fabrication, and characterization of a MEMS-based capacitive intraocular pressure (IOP) sensor for continuous wireless monitoring. The proposed battery-less implantable sensor shows strong potential for future integration within an FDA-approved minimally invasive glaucoma surgery (MIGS) device. The implantable LC IOP sensor and external interrogation circuit were systematically designed and optimized through multi-physics simulation and circuit analysis. A complete microfabrication and hermetic packaging process was developed to enable batch fabrication of a minimally invasive implantable sensor suitable for operation in the ocular environment. The results provide a foundation for ongoing and future in vivo studies involving implantation of a Parylene-encapsulated MEMS IOP sensor in the anterior chamber of a rabbit eye with an FDA-approved MIGS device. Wireless interrogation is performed by an external readout coil and a vector network analyzer or an impedance analyzer, while the development of portable point-of-care readout gadget is ongoing as our future work.

## Figures and Tables

**Table 1:** The comparison of the resistivity for the electroplated nickel and the bulk nickel.

Width (µm)	Total Length (µm)	Thickness (µm)	Sheet Resistance (Ω/□)	Resistivity (Ω·m)	Bulk Ni Resistivity (Ω·m)
20	10,050	2.057	0.0827	8.5 × 10^−8^	7.2 × 10^−8^

**Table 2:** The key design and performance parameters of an example IOP sensor design.

Parameter	Value	Unit
Inductance, *L_s_*	17.754	nH
Number of turns, *N*	3	-
Spiral inductor trace width, *w*	20	µm
Spiral inductor trace separation, *S*	20	µm
Spiral inductor outer diameter, *d_out_*	940	µm
Spiral inductor inner diameter, *d_in_*	740	µm
Capacitance, *C_s_*	9.32	pF
Capacitor electrode length, *l*	510	µm
Capacitor top-electrode thickness, *t*	2.057	µm
Parasitic capacitance, C_p_	1.3	pF
Measured resonant frequency, *f_0_meas_*	381.1	MHz
Quality factor, *Q*	1.104	-

**Table 3:** Comparison of implantable IOP sensors for different capacitive and inductive mechanisms.

Reference	Sensing Mechanism	Frequency (MHz)	*Q*	Device Size	Capacitance (pF)	Inductance (µH)	Sensitivity
This work	Capacitive	381.1	1.104	0.94 × 0.94 × 0.5 mm^3^	9.32	0.018	40 kHz/mmHg or 104 ppm/mmHg
[28]	Inductive	~350	~30	4 × 1.5 × 1 mm^3^	3.6	0.057	695 ppm/mmHg
[31]	Capacitive	103–95	8	2.6 mm × 1.6 mm	2.0–2.35	1.2	1553 ppm/mmHg
[36]	Capacitive	168	30	Diameter 2/4/6 mm, thickness 1/2 mm	-	2.5	-
[37]	Capacitive	~62	3	4 mm × 2 mm	8.5	0.78	7495 ppm/mmHg
[26]	Inductive	~63	26.5	2 × 2 × 0.8 mm^3^	5	1.3	15 kHz/mmHg
[38]	Capacitive	45	30	5 × 3 × 1 mm^3^	23	0.5	-
[39]	Capacitive	230	19.6	3.23 mm × 1.52 mm	0.93	0.515	3770 ppm/mmHg

## Data Availability

Measurements and other relevant data are available from the corresponding authors upon request via email.
